# Comparison of personality domains derived from personality ratings and behavioral observations in Japanese macaques (*Macaca fuscata*)

**DOI:** 10.1038/s41598-026-52385-y

**Published:** 2026-06-30

**Authors:** Kosho Katayama, Masayuki Nakamichi, Kazunori Yamada

**Affiliations:** Ethology, Graduate School of Human Sciences, The University of Osaka, 1-2 Yamadaoka, Suita, Osaka 565-0871 Japan

**Keywords:** Individual difference, Questionnaire, Primates, Japanese macaque, Personality assessment, Behavioral coding, Evolution, Neuroscience, Psychology, Psychology, Zoology

## Abstract

Primate personalities are a phenomenon of widespread interest. Researchers have attempted to measure primate personalities using two main methods: personality ratings and behavioral observations. However, a lack of consensus exists on whether the same personality domains can be measured using different methods. In our study, we aimed to explore the personality structure of 32 free-ranging Japanese macaques (*Macaca fuscata*) using personality ratings and behavioral observations. A principal component analysis of personality ratings assessed via questionnaires (the Hominoid Personality Questionnaire) revealed three components: Dominance_PR_, Anxiety_PR_, and Friendliness_PR_. Behavioral observations across a wide range of measures in daily situations also revealed two Behavioral Components we term Sociability_BC_ and Grooming_BC_. The behavioral domains captured individual tendencies in sociability, particularly with respect to the extent of interaction with kin. Comparison of the domains identified by the two methods showed that no overlapping domains were found. Furthermore, subject attributes such as rank and age were associated with Dominance_PR_ and Friendliness_PR_. This may be attributable to the lack of contextual information in the observational data. The concept of “personality coherence,” whereby behavior changes according to situational demands, may be important for understanding the association between personality ratings and behavioral observations.

**Supplementary Information:**

The online version contains supplementary file available at 10.1038/s41598-026-52385-y.

## Introduction

Consistent individual differences in behavior across time and situations among individuals of the same species are referred to as “personality” and have received attention from psychologists and biologists over the past three decades^1–6^. Personality usually appears as “domains” such as Dominance or Sociability. Researchers interpret these domains as being linked to the internal mechanisms of individuals^7^. Temporal stability and cross-situational consistency of behavior are prerequisites for establishing personality domains in human and non-human primates, and several studies on non-human primates have supported these assumptions^8–13^. Personality psychologists have long debated whether behavior shows less cross-situational consistency than previously thought^14–16^.

Researchers primarily quantify individual differences in non-human primate behavior using personality ratings^3,17–20^. In such psychological approaches, researchers typically aim to identify the primates’ comprehensive personality^21,22^. Researchers or zookeepers complete questionnaires about the personalities of subjects based on their long-term experience with and overall impressions of the primate subjects. The Hominoid Personality Questionnaire (HPQ) is based on the human Big Five or five-factor model and is frequently used to assess non-human primate personalities^3,23–25^. Although the HPQ has also been widely employed in studies of macaque species^6^, relatively few studies have focused on Japanese macaques (*Macaca fuscata*). The HPQ has been used to describe the personality structure of Japanese macaques from a provisioned group on Koshima Island, a wild population on Yakushima Island, and a zoo population in Italy resulting in four factors: Dominance, Openness, Friendliness, and Anxiety^26^. Substantial inter-rater reliability in HPQ assessments across different study sites suggests that Japanese macaques exhibit these four personality domains.

Some previous studies have also investigated the personality structures of primates through observational data alone. Behavioral observations can be used to assess personality by recording primate behavior using a predefined list of behaviors exhibited by the animal in its daily life, either in the wild^9,27–29^ or in its home enclosures in captivity^30,31^. With this method, personality structure is examined by repeatedly sampling behaviors expressed in everyday situations and identifying behavior patterns that remain stable throughout the observation period. For example, principal component analysis has confirmed that the behavioral data of wild crested macaques (*Macaca nigra*) reveal their personality structure through four factors: Anxiety, Connectedness, Sociability, and Aggressiveness^9^. Studies on various macaque species have reported species-specific differences in personality structures derived from behavioral data^31^. However, no study has examined this structure using observational data in Japanese macaques. Therefore, conducting behavioral observations in this relatively understudied species may further deepen our understanding of the selection pressures shaping primate personality.

Researchers have compared personality ratings and behavioral observations to examine whether questionnaire-based ratings comprehensively capture behavioral domains^30,32–39^. There is a lack of consensus on whether different methodologies can measure the same personality domains in primates^40–42^. For example, questionnaire-based ratings have been shown to correlate with specific behavioral measures in macaque species such as rhesus macaques (*Macaca mulatta*)^12,43^, Tibetan macaques (*Macaca thibetana*)^44^, and bonnet macaques (*Macaca radiata*)^37^, and long-tailed macaques (*Macaca fascicularis*)^42^. In Barbary macaques (*Macaca sylvanus*)^36^, partial correspondence has been found between rating components and behavioral component scores. A previous study on Assamese macaques (*Macaca assamensis*) discussed why different domains arose between personality ratings and behavioral observations^45^. Notably, macaques’ scores on Activity_TR_ (derived from ratings) strongly correlated with their scores on Sociability_BC_ (derived from behavioral observations), even though the two domains did not share semantically similar traits. Activity_TR_ included traits such as *playful*, *active*, *curious*, and *lazy*, whereas Sociability_BC_ consisted of traits such as *friendly behavior*, *contact diversity*, and *peripheral*, and did not include traits such as *active*. This association may have arisen because a primate’s “Activity” might have reflected “Social activity;” future research is needed to clarify these types of links^45^. Distinguishing behavioral measures of Sociability directed toward kin from those directed toward non-kin may help to capture a more Social active aspect of personality, beyond close connections with a limited number of familiar partners. In this way, predefined scale structures may not function as researchers expect, and different methodologies can yield different personality domains^30^. If questionnaire-based personality ratings truly capture consistent traits, they should reveal domains and correlates similar to those identified through behavioral observations. To validate personality ratings, empirical studies are required to test whether the same domains emerge in both personality ratings and behavioral observations across various daily situations.

We conducted a study on Japanese macaque personality. This species exhibits social structures based on dominance hierarchies, kinship, and age. For instance, grooming is usually directed toward higher-ranking individuals^46–48^. Grooming and proximity behaviors are more frequently found among kin than non-kin individuals^47–49^. Older female Japanese macaques focus their grooming on kin, independent of their dominance rank^50^. Thus, we considered dominance rank, number of kin, and age as variables that may affect behavioral expression. Although personality is often defined as consistent individual differences independent of dominance rank, kinship, and age, questionnaire-based ratings may still reflect biases^41^. For example, they might conflate sociability as a personality trait with a subject’s number of kin, leading to ratings that reflect both aspects, i.e., a monkey with many grooming partners may receive a high sociability rating simply due to its large number of kin. On the other hand, several studies suggest that links between personality scores and sex or age are not simply rating biases^20,26,31,51–53^. Japanese macaques offer a valuable model for testing whether questionnaire-based ratings can detect consistent behavioral domains while accounting for these variables.

In our study, we aimed to elucidate the personality structure of 32 free-ranging Japanese macaques using the 55-item HPQ^3,18,24^ and behavioral observations with a broad range of measures in daily situations. For our first hypothesis, we predicted that the HPQ would reveal domains similar to those identified in previous studies of Japanese macaques, including Dominance, Openness, Friendliness, and Anxiety/Reactivity^26,54^. Second, we hypothesized that the same personality structure could be found in the HPQ and behavioral observations. Specifically, we predicted that personality domains based on behavioral observations in daily situations would predict domains derived from personality ratings, even after controlling for sex, dominance rank, number of kin, and age. Because no study has identified domains from behavioral data in Japanese macaques, we conducted an exploratory investigation of a wide range of behaviors from activity to social behavior. To examine the potential link between social behavior and kinship, we assessed social behavior in two distinct forms: kin-related and non-kin-related. For instance, we divided grooming behavior into the proportion of time spent grooming kin and the proportion of time spent grooming non-kin.

## Results

### Personality structure of the ratings

We conducted personality ratings of 32 subject macaques (27 adult females and five adult males) using the 55-item HPQ. We excluded 28 of 55 items through the following stepwise exclusion process and finally selected 25 items as reliable measures to identify personality domains. First, inter-rater reliability was evaluated using *ICC*s (Table 1). Four of the 55 items with *ICCs* (3, k) < 0 were excluded as unreliable items^24^, and 51 items were retained. For the principal component analysis, the 31 items with the highest *ICCs* (3, k) were selected. Twenty items were excluded from further analysis because stable component estimation requires the number of items to be smaller than the number of subjects^55^, which was 32 in this study. Six items were sequentially excluded in order of increasing measures of sampling adequacy (MSA), starting with the items with the lowest MSA values, while maintaining an overall MSA of at least 0.6^9,56^: *dependent/follower*, *imitative*, *clumsy*, *individualistic*, *fond of infants*, and *active*. Finally, the 25 reliable items selected for analysis had a mean *ICC* (3, k) of 0.60 (SD = 0.06). The overall MSA was 0.61, indicating that the data were suitable for factor analysis.

**Table 1:** *ICC*s of the personality ratings (*N* = 32) of Japanese macaques in Japan in 2015.

Items	ICC (3, k)	95% CI	*p*
**Dominant**	0.94	[0.90, 0.97]	< 0.001
**Submissive**	0.80	[0.65, 0.89]	< 0.001
**Sociable**	0.73	[0.54, 0.86]	< 0.001
**Irritable**	0.71	[0.51, 0.85]	< 0.001
**Jealous**	0.70	[0.49, 0.84]	< 0.001
**Bullying**	0.69	[0.47, 0.83]	< 0.001
**Stable**	0.68	[0.45, 0.83]	< 0.001
**Manipulative**	0.68	[0.46, 0.83]	< 0.001
**Fearful**	0.66	[0.41, 0.82]	< 0.001
**Gentle**	0.66	[0.42, 0.82]	< 0.001
**Excitable**	0.66	[0.42, 0.82]	< 0.001
**Cool**	0.66	[0.42, 0.82]	< 0.001
**Aggressive**	0.65	[0.39, 0.81]	< 0.001
**Solitary**	0.63	[0.37, 0.80]	< 0.001
**Affectionate**	0.59	[0.30, 0.78]	< 0.001
**Timid**	0.54	[0.21, 0.76]	0.002
**Lazy**	0.52	[0.17, 0.74]	0.004
Dependent/Follower	0.50	[0.14, 0.73]	0.006
**Disorganized**	0.50	[0.15, 0.73]	0.005
**Persistent**	0.49	[0.13, 0.73]	0.007
**Stingy/Greedy**	0.48	[0.12, 0.72]	0.008
**Friendly**	0.43	[0.01, 0.69]	0.022
Clumsy	0.41	[− 0.01, 0.68]	0.028
Imitative	0.41	[− 0.01, 0.69]	0.026
**Quitting**	0.39	[− 0.05, 0.67]	0.038
Individualistic	0.38	[− 0.07, 0.67]	0.043
**Decisive**	0.35	[− 0.12, 0.65]	0.061
Fond of infants	0.35	[− 0.11, 0.65]	0.057
**Helpful**	0.32	[− 0.16, 0.64]	0.078
**Distractible**	0.31	[− 0.18, 0.63]	0.087
Active	0.30	[− 0.20, 0.63]	0.097
Unemotional	0.29	[− 0.21, 0.62]	0.103
Curious	0.27	[− 0.25, 0.61]	0.122
Innovative	0.27	[− 0.25, 0.61]	0.126
Unperceptive	0.27	[− 0.26, 0.61]	0.130
Sensitive	0.27	[− 0.25, 0.61]	0.126
Impulsive	0.25	[− 0.29, 0.60]	0.150
Intelligent	0.25	[− 0.28, 0.60]	0.145
Depressed	0.22	[− 0.33, 0.59]	0.177
Inventive	0.22	[− 0.34, 0.58]	0.180
Defiant	0.21	[− 0.35, 0.58]	0.191
Sympathetic	0.19	[− 0.38, 0.57]	0.214
Predictable	0.17	[− 0.42, 0.56]	0.240
Cautious	0.11	[− 0.52, 0.53]	0.324
Autistic	0.09	[− 0.56, 0.51]	0.352
Vulnerable	0.09	[− 0.56, 0.51]	0.354
Erratic	0.09	[− 0.56, 0.51]	0.352
Independent	0.09	[− 0.56, 0.51]	0.356
Thoughtless	0.04	[− 0.65, 0.49]	0.424
Playful	0.02	[− 0.67, 0.48]	0.447
Inquisitive	0.01	[− 0.70, 0.47]	0.464
Reckless	0.00	[− 0.72, 0.47]	0.480
Conventional	0.00	[− 0.72, 0.47]	0.480
Protective	0.00	[− 0.72, 0.47]	0.480
Anxious	0.00	[− 0.72, 0.47]	0.480
Mean	0.37		

Items retained in the principal component analysis are shown in bold.

We conducted a principal component analysis to detect the personality domains of Japanese macaques using the 25 items with high inter-rater reliability for our 32 subjects rated by three raters. The three raters did not include the author (K. K.) who conducted the behavioral observations. This reduced the possibility that the personality ratings merely reflected the author’s subjective evaluations. Visual inspection of the scree plot revealed three components, and parallel analysis also suggested three components. The eigenvalues exceeded 1.00 for the five components. We extracted three components in the personality ratings. The mean absolute value of correlations between the varimax- and promax-rotated principal component scores of the three components was high (*r* = 0.98, SD = 0.01), indicating that the component structure was similar across rotation methods. We interpreted the personality structure and labeled the three components of personality ratings (PRs) as Dominance_PR_, Anxiety_PR_, and Friendliness_PR_ (Table 2). Dominance_PR_ showed positive loadings for items such as *persistent*, *stingy/greedy*, and *dominant*, and negative loadings for items such as *submissive*, *fearful*, and *timid*. Anxiety_PR_ showed positive loadings for *excitable*, *aggressive*, and *irritable*, and negative loadings for *gentle*, *cool*, and *stable*. Friendliness_PR_ showed positive loadings for *helpful*, *friendly*, and *affectionate*, and negative loadings for *lazy* and *solitary*. These three components accounted for 72% of the total variance. The mean Cronbach’s α across the three components was 0.90 (SD = 0.04). We calculated their principal component scores based on the promax solution, and computed the correlation between the components (Supplementary File 1).

**Table 2:** Factor loadings, sum of squared loadings, cumulative contribution, mean Cronbach’s α and *ICC* (3, k) of three components based on personality ratings (*N* = 32) of Japanese macaques in Japan in 2015.

Items	Components		
	Dominance_PR_	Anxiety_PR_	Friendliness_PR_
Persistent	**0.90**	0.05	− 0.28
Stingy/greedy	**0.88**	0.24	− 0.12
Dominant	**0.86**	0.14	0.16
Submissive	**− 0.84**	− 0.05	− 0.13
Jealous	**0.84**	0.17	0.13
Fearful	**− 0.79**	0.33	0.02
Timid	**− 0.72**	0.13	− 0.27
Bullying	**0.55**	**0.67**	0.21
Excitable	0.04	**0.92**	0.01
Aggressive	0.33	**0.90**	0.16
Irritable	0.15	**0.90**	− 0.01
Gentle	− 0.13	**− 0.85**	0.23
Distractible	− 0.01	**0.85**	− 0.07
Cool	0.05	**− 0.81**	0.20
Stable	0.33	**− 0.80**	0.11
Lazy	**0.47**	**− 0.60**	**− 0.73**
Sociable	**0.44**	0.08	**0.72**
Solitary	**− 0.49**	− 0.09	**− 0.65**
Decisive	**0.45**	− 0.17	**0.49**
Helpful	0.06	− 0.04	**0.78**
Friendly	− 0.14	− 0.26	**0.77**
Affectionate	0.13	− 0.29	**0.60**
Disorganized	0.39	0.18	**− 0.49**
Manipulative	0.13	0.04	**0.49**
Quitting	− 0.38	0.13	− 0.27
Sum of squared loadings	6.64	6.57	4.57
Cumulative contribution	0.28	0.54	0.72
Cronbach’s α of salient Items	0.94	0.93	0.84
Mean *ICC* (3, k) of salient items	0.63	0.66	0.53

Salient loadings ≥ |0.4| are shown in bold.

### Personality structure of the behavioral measures

We extracted the personality domains of the behavioral observations from nine temporally stable behavioral measures identified through repeatability (R) analysis^57^. These nine behavioral measures showed moderate to high repeatability (*R* > 0.2)^58^ and were therefore temporally stable behavioral measures (Table 3). Eleven measures with low repeatability were excluded from further analysis. To achieve an overall MSA greater than 0.6, two variables with the lowest MSA values were removed from the analysis. As a result, *prop time spent moving* (MSA = 0.38) and *rate receiving supplanting behavior* (MSA = 0.26) were excluded. Finally, seven behavioral measures were selected for the component analysis, and a z-transformation was applied to these variables. The mean repeatability of the seven retained measures was 0.29 (range: 0.22–0.46), suggesting that these seven measures showed moderate repeatability (0.2 < *R* < 0.4) on average over two years^58^. The overall MSA was 0.61, indicating that the data were suitable for factor analysis. Two components had eigenvalues greater than 1.00. An examination of the scree plot suggested there were two components, and parallel analysis indicated two components. We extracted two components and calculated their principal component scores based on the promax solution, and computed the correlation between the components (Supplementary File 2). We labeled the two behavioral components (BCs) as Sociability_BC_ and Grooming_BC_ (Table 4). Sociability_BC_ showed positive loadings for *grooming kin* and *approaching kin*, and negative loadings for *sitting* and *scratching*. Grooming_BC_ showed positive loadings for *grooming non-kin* and *the number of non-kin grooming partners*. Although only two measures loaded on the Grooming_BC_, final components with few items are common in previous studies^9,27–29,31,32,44^; thus, we retained two components. These two components accounted for 69% of the total variance. The mean Cronbach’s α across the two components was 0.86.

**Table 3:** Repeatability of behavioral measures (*N* = 32) after controlling for sex, dominance rank, age, and number of kin in Japanese macaques in Japan from 2013 to 2015.

Behavioral measure	Repeatability	95% CI	*p*
**Rate approaching kin**	0.46	[0.00, 0.64]	0.022
Prop time spent moving	0.40	[0.00, 0.62]	0.010
**The number of non-kin grooming partners**	0.32	[0.00, 0.47]	0.064
**Prop time spent sitting**	0.32	[0.00, 0.54]	0.038
**Prop time spent grooming non-kin**	0.25	[0.00, 0.53]	0.116
**Prop time spent grooming kin**	0.23	[0.00, 0.53]	0.141
Rate receiving supplanting behavior	0.23	[0.00, 0.48]	0.150
**Rate scratching**	0.23	[0.00, 0.48]	0.108
**Prop time spent in proximity with kin**	0.22	[0.00, 0.55]	0.156
Rate threat	0.19	[0.00, 0.45]	0.179
The number of non-kin proximity partners	0.12	[0.00, 0.39]	0.258
Prop time spent self-grooming	0.11	[0.00, 0.42]	0.270
Prop time spent in proximity with non-kin	0.08	[0.00, 0.37]	0.327
Rate approaching non-kin	0.05	[0.00, 0.32]	0.393
Prop time spent lying down	0.03	[0.00, 0.36]	0.435
Prop time spent feeding	0.00	[0.00, 0.28]	0.500
Rate aggression	0.00	[0.00, 0.32]	0.500
Rate supplanting behavior	0.00	[0.00, 0.28]	1.000
The number of kin grooming partners	0.00	[0.00, 0.16]	1.000
The number of kin proximity partners	0.00	[0.00, 0.13]	0.500
Mean	0.16		

Items retained in the principal component analysis are shown in bold.

**Table 4:** Factor loadings, sum of squared loadings, cumulative contribution, mean Cronbach’s α and repeatability of two components based on behavioral observations (*N* = 32) of Japanese macaques in Japan from 2013 to 2015.

Behavioral measure	Components		
	Sociability_BC_	Grooming_BC_	h^2^
Prop time spent sitting	**− 0.83**	− 0.19	0.78
Prop time spent grooming kin	**0.81**	− 0.14	0.64
Rate approaching kin	**0.80**	0.22	0.76
Prop time spent in proximity with kin	**0.75**	− 0.02	0.55
Rate scratching	**− 0.45**	0.37	0.28
Prop time spent grooming non-kin	0.07	**0.93**	0.89
The number of non-kin grooming partners	0.13	**0.92**	0.90
Sum of squared loadings	2.81	1.99	
Cumulative contribution	0.40	0.69	
Cronbach’s α of salient measures	0.78	0.93	
Mean repeatability of salient behavioral measure across two time periods	0.29	0.28	

Salient loadings ≥ |0.4| are shown in bold.

### Comparison of personality structure between the personality ratings and behavioral observations

We examined whether the same personality structure could be found in the HPQ and behavioral observations using linear regression models of 32 subjects (Table 5). Three models were used to examine the effects of sex, dominance rank, the number of kin, age, and component scores of Sociability_BC_, and Grooming_BC_ on each of the three response variables: Dominance_PR_, Anxiety_PR_, and Friendliness_PR_. Multicollinearity diagnostics indicated high variance inflation factors (VIFs > 3) for number of kin (VIF = 3.7) and Sociability_BC_ (VIF = 3.8). The mean VIF of the total six predictors was 2.3 (range: 1.3–3.8). Because the number of kin was considered an important environmental variable affecting individuals in this study, and the variance inflation factor was below 10, we retained this variable in the models. To verify model validity, we conducted residual diagnostics, and in all three models, the outlier test, the dispersion test, and the Kolmogorov–Smirnov (uniformity) test were non-significant (Supplementary File 3). The plot of residuals against predicted values did not detect any notable problems. These results indicate that we appropriately modeled the relationships between the explanatory and the response variables.

**Table 5:** Results of three linear regressions with rating domains as response variables (*N* = 32) for Japanese macaques in Japan from 2013 to 2015.

Response variables	Explanatory variables	Estimate	SE	95% CI	t	*P*
Dominance_PR_	(Intercept)	− 0.01	0.12	(− 0.20, 0.18)	− 0.08	0.940
	Sex (male)	0.06	0.39	(− 0.59, 0.70)	0.14	0.888
	Rank	− 0.78	0.13	(− 0.99, − 0.57)	− 6.11	**0.000**
	Age	0.26	0.12	(0.07, 0.45)	2.22	**0.036**
	Number of kin	− 0.12	0.19	(− 0.44, 0.19)	− 0.63	0.534
	Sociability_BC_	0.26	0.20	(− 0.07, 0.58)	1.31	0.203
	Grooming_BC_	0.12	0.11	(− 0.07, 0.31)	1.05	0.302
Anxiety_PR_	(Intercept)	0.07	0.21	(− 0.27, 0.40)	0.33	0.746
	Sex (male)	− 0.43	0.69	(− 1.57, 0.71)	− 0.62	0.542
	Rank	− 0.26	0.22	(− 0.63, 0.11)	− 1.17	0.255
	Age	− 0.34	0.21	(− 0.68, − 0.00)	− 1.66	0.109
	Number of kin	− 0.23	0.34	(− 0.79, 0.32)	− 0.69	0.497
	Sociability_BC_	− 0.02	0.34	(− 0.59, 0.54)	− 0.07	0.947
	Grooming_BC_	0.21	0.20	(− 0.12, 0.54)	1.07	0.295
Friendliness_PR_	(Intercept)	0.16	0.16	(− 0.11, 0.43)	1.00	0.325
	Sex (male)	− 1.05	0.55	(− 1.97, − 0.14)	− 1.90	0.069
	Rank	− 0.60	0.18	(− 0.90, − 0.31)	− 3.35	**0.003**
	Age	− 0.19	0.17	(− 0.46, 0.08)	− 1.15	0.262
	Number of kin	0.33	0.27	(− 0.12, 0.78)	1.22	0.234
	Sociability_BC_	0.07	0.28	(− 0.38, 0.53)	0.27	0.793
	Grooming_BC_	0.00	0.16	(− 0.26, 0.27)	0.03	0.980

Statistically significant results are shown in bold.

We did not find a consistent association between personality ratings and behavioral observations in our study. We found a negative association between Dominance_PR_ and dominance rank, such that individuals with lower numerical rank values (i.e., higher-ranking subjects) had higher Dominance_PR_ scores. Age was positively associated with Dominance_PR_. Friendliness_PR_ was negatively associated with dominance rank, indicating that higher-ranking subjects had higher Friendliness_PR_.

## Discussion

This study provides a comprehensive assessment of personality in Japanese macaques based on personality ratings and behavioral observations. A principal component analysis of HPQ ratings revealed three components: Dominance_PR_, Anxiety_PR_, and Friendliness_PR_. Behavioral observations also yielded two components: Sociability_BC_ and Grooming_BC_. To examine the similarities between the rating-derived and behavior-derived components, the three rating-based personality components were used as response variables, whereas sex, dominance rank, age, number of kin, and the component scores of the two behavioral components Sociability_BC_ and Grooming_BC_ were included as explanatory variables in the GLMs. We did not find an association between personality ratings and behavioral observations in our study. In addition, higher-ranking subjects had higher Dominance_PR_ and Friendliness_PR_, and older subjects had higher Dominance_PR_ scores.

Regarding the initial hypothesis, the results partially support our predictions. The three components closely resemble those reported in previous studies of Japanese macaques, although some differences were observed between the components. Our first domain, Dominance_PR_, corresponds to the Dominance commonly found in other non-human primate species in the context of HPQ ratings^2,24,54^. Dominance_PR_ in our study included the same traits as the Dominance factor reported in previous studies of Japanese macaques^26,54,59^, namely *persistent*, *stingy/greedy*, *dominant*, *submissive*, *fearful*, and *timid*. In particular, Assamese macaques^26,45^, Barbary macaques^20,26,36^, crested macaques, Tonkean macaques (*Macaca tonkeana*)^26^, and rhesus macaques^24^ all share a common domain that includes *dominant*, *submissive*, *fearful*, and *timid*, suggesting that this domain may be widely shared across macaques.

Anxiety_PR_ in our study and Anxiety/Reactivity in a previous study of Japanese macaques^54,59^ likely represent the same domain, as both include the items *excitable*, *aggressive*, *irritable*, *gentle*, and *stable*. In addition, Anxiety_PR_ in our study shared at least five of its nine traits (i.e., *aggressive*, *bullying*, *excitable*, *gentle*, and *irritable*) with domains identified in other macaque species, including Aggressiveness in crested macaques^26^, Dominance in rhesus macaques^24^, and Opportunism_TR_ in Assamese macaques^45^, suggesting that these domains may be similar. Despite this overlap, the two components were labeled differently. Some researchers have noted that such inconsistencies may reflect “jingle-jangle fallacies” stemming from differences in assessment methods^30,60^. In contrast, the traits *cool*, *distractible*, and *lazy* in Anxiety_PR_ in our study were not shared with most other macaque species and may have been traits specific to the subjects in this study.

Friendliness_PR_ expressed how actively one individual tried to interact with the other monkeys in the group. Friendliness_PR_ in this study was similar to that in the previous study in that it included the traits *affectionate*, *solitary*, and *friendly*^54,59^. Friendliness_PR_ in our study, which included *affectionate*, *friendly*, *helpful*, *sociable*, and *solitary*, shared traits with Friendliness in another group of Japanese macaques, as well as with Friendliness in crested macaques^26^, Barbary macaques^20,26,36^, rhesus macaques^24^, Tonkean macaques^26^, and Assamese macaques^26,45^, suggesting that Friendliness may be a domain broadly shared across macaque species. On the other hand, the Friendliness domain in a previous study of Japanese macaques included the traits *excitable*, *irritable*, *gentle*, and *stable*^26^. Because Japanese macaques differ greatly in tolerance across groups^61^, the expression of these personality domains may vary based on different raters and habitats. Future research should clarify whether Friendliness varies depending on context, i.e., whether the monkeys live in the wild or in captivity, whether their behavior is directed toward other monkeys or humans, and whether the raters are researchers or zookeepers.

**Fig. 1:**
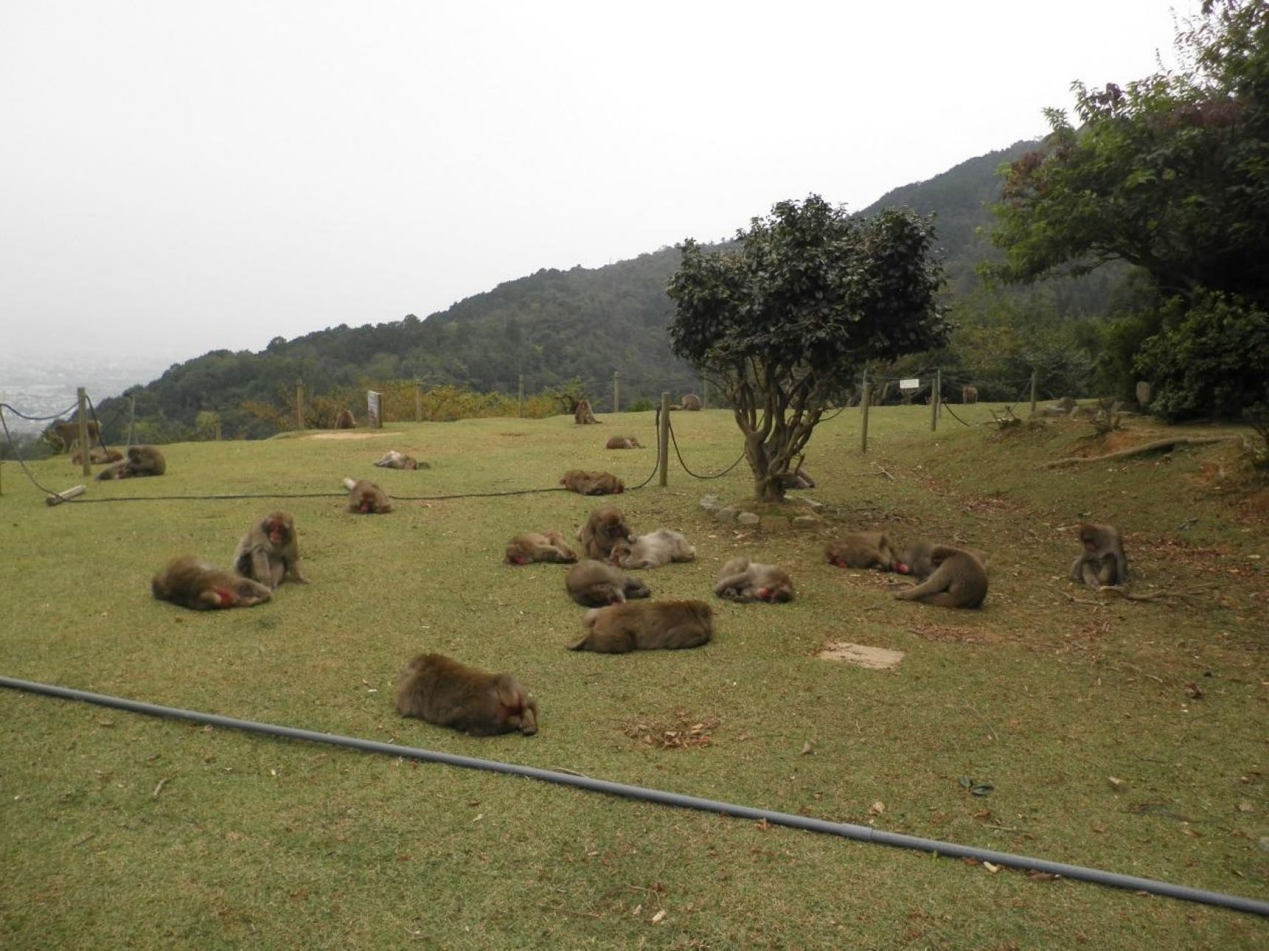
Kin individuals frequently gathered and rested together during the observation period. The focal animal sampling method captured this type of monkey behavior as behavioral components Sociability_BC_ (staying in proximity to kin partners). The photograph was taken by the first author, K. K.

The results of the principal component analysis of the behavioral observations were similar to those of previous studies^48,62^. Sociability_BC_ in our study may have been similar to Sociability_BC_ in Assamese macaques^45^, which included *friendly behavior*, *peripheral*, *contact time*, and *self-directed behavior*. Sociability_BC_ in our study may reflect a subject’s tendency to frequently associate with kin individuals and to rest (i.e., sitting). We frequently observed higher-ranking kin individuals gathering for rest and lower-ranking subjects walking around the group during the observation period (Fig. 1). Previous studies have shown that higher-ranking individuals tend to stay near the provisioning site, whereas lower-ranking individuals remain in peripheral areas^62,63^. Sociability_BC_ in this study, at least in part, may reflect a subject’s tendency to frequently approach and spend time in proximity to kin. This might align with previous findings suggesting that Sociability may not necessarily reflect a standalone domain, but may instead capture “social activity” in group-living primates^45^.

Grooming_BC_ reflects a tendency to form diverse grooming relationships with non-kin individuals. Previous studies have shown that macaques develop close relationships with non-kin individuals, even if there are kin in the group^48,62^. In addition, Grooming_BC_ shows similarities to the Sociability domain described in crested macaques, as that component includes behavioral measures such as *diversity grooming partner*^9^. A grooming-related behavioral domain has also been identified in Barbary macaques (Tactility_BC_, including *allogroom*, *neighbors within 1 m*, and *grooming density*)^36^. In contrast, no such grooming-related domain involving interactions with other individuals has been reported in captive long-tailed macaques, pigtailed macaques (*Macaca nemestrina*), or rhesus macaques, suggesting that habitat may influence the expression of these domains^31^. The main difference between this and the previous study lies in the inclusion of kin interactions as part of the Sociability measure. Our analysis revealed Sociability_BC_ (interaction with kin) and Grooming_BC_ (interaction with non-kin) to be independent domains. This suggests that some Japanese macaques frequently interact with non-kin regardless of how much they associate with kin. In this study, we found temporally stable individual differences even after controlling for individual attributes (i.e., sex, dominance rank, number of kin, and age). Behavioral tendencies related to Sociability have also been proposed in the context of behavioral syndromes^7^ and may reflect individual differences in social strategies.

Contrary to our second hypothesis, a comparison of the personality structures of Japanese macaques in personality ratings and behavioral observations revealed that different personality structures were found using the two methods. We identified two behavioral domains, Sociability_BC_ and Grooming_BC_, but neither predicted rating-based Friendliness_PR_. This may be because the observational data lacked contextual information. Although focal observations were conducted across a wide range of everyday situations, the same behavior may reflect different underlying processes depending on the context; for example, grooming may occur as reconciliation after an aggressive interaction^64^. Personality traits may differ in their degree of “visibility,” making some domains easier to rate than others^65,66^. The idea of “personality coherence,” in which behavior changes according to situational demands^67,68^, may be important for understanding the association between personality ratings and behavioral observations. In addition, behavioral observations, conducted by a single observer, assessed inter-individual consistency across two periods, whereas personality ratings assessed reliability across multiple raters. Although both are commonly used to measure personality^6^, they may not capture exactly the same aspect of it. It will also be important for future studies to examine the temporal stability of personality ratings.

HPQ ratings were associated with individual attributes such as dominance rank and age. Higher-ranking individuals had higher Dominance_PR_, which may be consistent with previous findings that rank is associated with Confidence in Barbary macaques^20^. Higher rank was also associated with higher Friendliness_PR_, which may be attributable to the correlation between Dominance_PR_ and Friendliness_PR_ (Supplementary File 1). HPQ ratings may partly reflect the influence of individual attributes such as dominance rank and age^20,23,30,39,52^. On the other hand, several studies have shown that associations between age, rank, and personality cannot be explained solely by rating biases^20,26,31,51–53^. Future studies should account for these attributes in HPQ ratings, which may clarify the relationship between personality ratings and behavioral measures.

Our study has three methodological limitations. The first limitation concerns the short observation time. The total observation time per subject (6.6 h) was relatively large compared with that in recent macaque studies, in which observation times per subject ranged from just over 1 h to less than 6 h^30,39,69,70^. Previous research has suggested that more than 5 h of observation is sufficient to obtain stable personality factors^66^; the observation time in this study was adequate for analyzing personality domains. However, in our study, we divided the observation period into two periods (Period 1 and Period 2) and calculated repeatability across them. The number of observation days and the total observation time differed between the two periods, which may have affected the estimation of repeatability for stable behavioral measures. Future studies should examine this issue under more strictly controlled observation periods and durations.

The second limitation concerns the age composition of the subjects, which may explain the absence of Openness-related features in the component analysis. Although we selected subjects from adult females spanning a wide age range, 48 of the 88 adult females in the study group (> 6 years old) were 20 years old or older at the beginning of the study. Thus, the average age of the subjects was relatively high at 21.38 years (SD = 4.09; age range: 13–31 years). In other primate species, younger individuals tend to display higher levels of Openness^23,52^ or *playfulness*^30,39^. Therefore, domains such as Activity and Openness, common domains across macaque species^24^, may vary depending on group age composition. In addition, older subjects tended to have higher rank (*r* = −0.37), which may partly explain why older subjects had higher Dominance_PR_ scores. Future studies should consider the age composition of the subject group when these domains are examined.

The third limitation is the small sample size. Our study included only 32 subjects, and this constraint required us to reduce the number of personality-rating items. A larger sample would have allowed the assessment of a broader range of trait ratings. In addition, the sample may not be sufficient to generalize the findings to Japanese macaque personality more broadly. Our study was conducted in a provisioned group at a field site visited by tourists. Although we could not examine whether the presence of visitors influenced macaque personality in this study, future comparisons with other wild groups may help to address this issue.

In conclusion, personality ratings revealed three personality domains in Japanese macaques, and these domains were similar to those identified in previous studies. Behavioral observations identified two domains related to Sociability. Comparison of the two methods showed that personality ratings and behavioral observations did not yield similar personality structures. Rather than relying solely on broad sampling across everyday situations, future research may better clarify the relationship between these two methods by taking into account the context-dependent expression of personality.

## Methods

### Ethical statement

All observations were conducted in accordance with the Regulations on Animal Experimentation at Osaka University, Japan, and approval was obtained from the Animal Research Committee of the Graduate School of Human Sciences, Osaka University (No. 24-2-0). Our research also followed the ARRIVE guidelines^71^ and the American Society of Primatologists (ASP) Principles for the Ethical Treatment of Nonhuman Primates and Code of Best Practices in Field Primatology and complied with all applicable national laws. We carried out observations at the privately owned Arashiyama Monkey Park in Kyoto, Japan, with permission from the site’s management. All raters provided their free, prior, and informed consent to participate in the study.

### Study site and subjects

The study subjects were 32 Japanese macaques (Supplementary File 4 and 5) from a free-ranging, provisioned group (the Arashiyama group) living in Arashiyama Monkey Park (35°00′N, 135°40′E), Arashiyama, Kyoto, Japan. The macaques were monitored by staff to prevent direct interactions with tourists. In addition, although the study subjects were also observed by other researchers, no experimental studies were conducted on them. We selected 32 adult subjects with as much variation as possible in age, maternal lineage, and rank. Including the preliminary observation period, the overall study was conducted between August 2013 and October 2015. In August 2013, the group comprised 126 individuals, including ten adult males (≥ 5 years old), 90 adult females (≥ 5 years old), 19 immatures (aged 1–4 years), and seven infants (aged < 1 year old). In October 2015, the group comprised 113 individuals: seven adult males (≥ 5 years old), 87 adult females (≥ 5 years old), 12 immatures (1–4 years old), and seven infants (< 1 year old). Maternal kin relationships and the ages of individuals in the group have been well documented since the introduction of provisioning in 1954^72–74^. We defined maternal kin as individuals with a k degree of relatedness of 0.25 or higher. Mother–offspring, sibling, and grandmother–grandchild relationships qualified as kin. Before the data were collected, we identified all group members, including infants, based on their facial features, body characteristics, and behavior. In the Arashiyama group, the breeding season is from late September to March, and the birth season is from April to August^75^.

### Personality ratings and behavioral observations

We conducted personality ratings on our subject macaques using the HPQ (Supplementary File 6–10). Between October and November 2015, we rated 27 adult females (mean age = 20.93 ± 3.52 years) and five adult males (mean age = 23.80 ± 5.49 years). The HPQ originally consisted of 54 items scored on a seven-point Likert scale^24,26^, using the Japanese-translated version identical to the version used in a previous study in Japan^18^. Because preliminary observations indicated individual variation in approaching behavior toward conspecifics with infants, we added *fond of infants* as an additional sociability item, bringing the total to 55 items. Five researchers with at least two years of experience with the group provided personality ratings. To prevent bias, all raters received instructions to refrain from discussing their ratings. One researcher failed to complete the questionnaire, so the analysis included ratings from four raters, including the author (K. K.). All four raters were able to identify all subjects based on facial or behavioral characteristics.

We conducted behavioral observations using the focal animal sampling method^76^(Supplementary File 11). The first author (K. K.) collected behavioral data with 20-min focal sessions from 0900 to 1730 h, when most macaques resided near the provisioning site. The subjects were observed in a predetermined random order. The observer recorded 20 social and non-social behaviors occurring during the daily routine of the Arashiyama group to capture a broad spectrum of activities, using either an instantaneous sampling method with 30-second intervals or an all-occurrence sampling method (Table 6). A wristwatch with an alarm (CASIO DW5600-1) and sampling sheets facilitated data recording. If the observer could not find the focal subject, they observed the next subject in the predetermined order. Whenever the observer missed a subject in the scheduled order, they began observing the subject as soon as it was found in the park. The observer identified an estrus session when the focal subject was in estrus and recorded it if the subject exhibited any of the following: having a redder face than individuals outside the estrus season, having the presence of semen or a copulatory plug on the rear end, emitting estrus calls, being mounted by males, or mounting other individuals.

**Table 6:** Behavioral measures of Japanese macaques in Japan from 2013 to 2015.

Behavioral measures	Description	Method
Prop time spent moving	Proportion of point samples out of all point samples spent moving over body length or standing on four legs	Instantaneous
Prop time spent sitting	Proportion of point samples out of all point samples spent with time in which nothing was done except sitting	Instantaneous
Prop time spent lying down	Proportion of point samples out of all point samples spent with time in which nothing was done except lying down	Instantaneous
Prop time spent feeding	Proportion of point samples out of all point samples spent feeding	Instantaneous
Prop time spent self-grooming	Proportion of point samples out of all point samples spent self-grooming	Instantaneous
Rate scratching	Hourly rate of scratching its body twice or more consecutively	All-occurrence
Rate threat	Hourly rate of threats directed at other individuals/receiving threats. Threats directed at people such as tourists, staff, and an observer were also recorded	All-occurrence
Rate aggression	Hourly rate of aggression such as biting, jumping on, and chasing other individuals/receiving aggression. Approaching with threats directed at people such as tourists, staff, and an observer were also recorded	All-occurrence
Rate supplanting behavior /receiving supplanting behavior	Hourly rate of moving another individual away from its original location when the subject approaches another individual without apparent agonistic behavior between two individuals/receiving supplanting behavior	All-occurrence
Prop time spent grooming kin/non-kin	Proportion of point samples out of all point samples spent grooming kin/non-kin	Instantaneous
The number of kin/non-kin grooming partners	The total number of subject’s kin/non-kin that subjects groomed during time spent grooming kin/non-kin. Multiple interactions with same individual were recorded as one	
Prop time spent in proximity with kin/non-kin	Proportion of point samples of spending proximity with kin/non-kin (within 3 m, arm-reach, and contact) out of all point samples	Instantaneous
The number of kin/non-kin proximity partners	The number of individuals with which subjects have been in proximity (within 3 m, arm-reach, and contact) during time spent in proximity with kin/non-kin. When the subjects spent with the same individual multiple times, it was counted as one	
Rate approaching kin/non-kin (3 m and arm-reach)	Hourly rate of moving within a range of 3 m of other individuals and staying for more than 2 s, or receiving approaching from other individuals	All-occurrence

Excluding estrus sessions, the total number of sessions was 634.80 (mean = 19.84 ± 1.84), and the total behavioral observation time was 211.60 h for 32 individuals (mean = 6.61 ± 0.61 h). The total observation time per subject in this study (6.6 h) was relatively large compared with that of recent macaque studies, in which observation times per subject range from more than one hour to less than six hours^30,39,69,70^. Given that previous research indicates that more than five hours of observation is sufficient to obtain stable personality factors^66^, the observation time in this study was adequate for analyzing personality domains. The total number of observation days between November 2013 and October 2015 was 119. We conducted an average of 3.10 observation sessions per day (SD = 1.59). The observation period of this study consisted of two periods: Period 1 and Period 2. Period 1 extended from November 20, 2013, to March 28, 2015, and included 89 observation days. During this period, a total of 391.61 sessions were conducted (mean per subject = 12.24 ± 1.48), yielding a total observation time of 130.43 h (mean per subject = 4.08 ± 0.50 h). The average interval between observation days was 5.60 days (SD = 4.55). The interval between Period 1 and Period 2 was 170 days. Period 2 extended from September 14, 2015, to October 30, 2015, and included 30 observation days. During this period, a total of 243.16 sessions were conducted (mean per subject = 7.60 ± 1.35), yielding a total observation time of 80.92 h (mean per subject = 2.53 ± 0.45 h). The average interval between observation days was 1.59 days (SD = 1.02).

The dominance rank of each individual was based on the knowledge of park staff and N. K. (unpublished data). These ranks were determined using dominance-related behaviors such as aggression, threats, supplantings, and submissive facial and vocal signals recorded during both focal animal sampling and ad libitum observations. Female dominance rank was calculated based on 1,263 dominance-related behaviors recorded between April 2013 and March 2014. From these data, we constructed a dyadic win–loss matrix and calculated female dominance rank using David’s scores^77^ implemented in the R package “steepness”^78^(Supplementary File 4). Because no dominance-related behaviors were recorded for the focal individual H99, its David’s score was 0. However, park staff and researchers were aware that this individual was the lowest-ranking member of the group. Therefore, individuals for whom dominance-related behavior could be recorded were assigned ordinal dominance ranks from 1 to 89, with 1 indicating the highest rank and 89 the lowest. During the observation period from August 2013 to October 2015, three adult females disappeared from the group. Dominance rank in Japanese macaques in the Arashiyama group tends to remain stable over time^79^. In addition, researchers monitored the group and the movements of central males on a daily basis, and would therefore have been able to detect any major changes in dominance rank; thus, we confirmed that no substantial rank changes occurred during the study period.

### Analysis of personality ratings

We screened items using the following three steps to identify reliable personality domains. First, we assessed the inter-rater reliability of each questionnaire item using the scores from the 32 macaques. We calculated two types of intraclass correlation coefficients^80^: *ICC* (3, 1) for single-rater agreement and *ICC* (3, k) for multiple-rater agreement. Following the previous study, we excluded items with *ICC*s below zero^24^. Second, because stable component estimation requires the number of items to be smaller than the number of subjects^55^, the number of items was reduced to 31, as the number of subjects in this study was 32. Third, following previous studies^9^, we calculated the Kaiser-Meyer-Olkin (KMO) measure of sampling adequacy and removed items with a low measure of sampling adequacy (MSA) to ensure that the overall MSA was greater than 0.6^9,56^.

We began the analysis by calculating individual item scores by averaging ratings across raters, followed by standardizing all item scores using z-transformation. To determine the number of components, we examined the scree plot and performed a parallel analysis^81^. We used the principal function from the “psych” package^82^ and applied the promax rotation to facilitate component interpretation. We calculated component scores and evaluated whether the domain structure varied across rotation methods by examining the correlations between the component scores derived from the varimax and promax rotations. We labeled each component based on items with salient loadings (≥ 0.40) in reference to previous studies on macaque personality^10,12,24,26,54^. Although salient cross-loadings are commonly treated as grounds for item revision or removal in exploratory factor analysis, the literature does not regard their exclusion as an absolute requirement^20,23,24,37,45,83^; therefore, we retained the cross-loading items. To examine whether the questionnaire items constituting each component served as reliable indicators of that component, Cronbach’s alpha was calculated for the items with salient loadings.

### Analysis of behavioral observations

We investigated whether the 20 behavioral measures were stable over time using repeatability^57^. Repeatability represents consistent inter-individual differences in behavior and is defined as the proportion of the total variance (i.e., intra- plus inter-individual variance) attributable to inter-individual differences. To estimate repeatability, we used raw behavioral codings and calculated the repeatability across the two observation periods, Period 1 and Period 2. Calculating consistency over a period of up to two years by dividing the observation period into two or three time blocks is a common approach in studies investigating personality through behavioral observation^9,36,45,69,70^. Repeatability was estimated using Poisson models, with sex, dominance rank, age, and number of kin included as fixed effects. To control for differences in observation effort between Period 1 and Period 2, observation time or the number of observation points was included as an offset term. Repeatability (R) is often classified into three categories^58^: low (*R* ≤ 0.2), moderate (0.2 < *R* < 0.4), and high (*R* ≥ 0.4). We retained behavioral measures with repeatability ≥ 0.2 for the principal component analysis, adopting a relatively lenient cutoff. Repeatability calculations used the “rptR” package in R^57^.

We then conducted a principal component analysis to detect personality domains from temporally stable behavioral variables with high repeatability. This analysis involved the summation of behavioral data and calculation of proportions of time from the instantaneous sampling and frequencies from all-occurrence sampling over the observation period. We calculated the KMO and removed behavioral measures with MSA values below 0.4 to ensure that the overall MSA exceeded 0.6. All the behavioral measures underwent z-transformation before the parallel analysis. Scree plots and parallel analysis guided the determination of the number of components, and the promax rotation was used to extract the components, following the same procedure used for the personality ratings. Cronbach’s alpha was computed for behavioral measures with salient loadings after the principal component analysis was completed.

### Comparison of personality structure between the personality ratings and behavioral observations

In our analysis, we tested whether the personality structure derived from ratings corresponded to that derived from behavioral observations. To avoid conceptual circularity, we modeled behavior as influencing the ratings. Linear regression with a Gaussian error distribution (identity link) was used to evaluate the effects of sex, dominance rank, number of kin, age, and scores of behavioral components on each personality rating score. Principal component scores for the behavioral and rating components were computed in R using the “psych” package^82^ from standardized variables, with regression-based weights derived from the rotated structure matrix after promax rotation. All the variables were continuous, with the exception of sex, and continuous measures were scaled and centered. Each model included the same set of predictors to allow for explanatory analysis, as the behavioral domains have not been clearly identified in Japanese macaques. The glm function from the “stats” package^84^ was used to carry out the analyses.

We checked for multicollinearity among the predictors using VIFs. Although a VIF threshold of 3 has been used as a stricter criterion^85^, a cutoff of 10 or less is also widely used^86^. To assess model validity, we conducted a dispersion test, an outlier test, and a uniformity test using the “DHARMa” package^87^, and further diagnosed residual variance by inspecting the plot of residuals against predicted values^88^. Statistical significance was determined using a threshold of *p* = 0.05.

In this analysis, the raters did not collect any behavioral data. The principal component scores were based on the ratings provided by three raters, excluding the first author (K. K.), who conducted the behavioral observations. This reduced the possibility that the personality ratings simply reflected the evaluations of the author. We additionally conducted a principal component analysis using ratings from four raters (including the first author, K. K.) and confirmed that the component structure was similar between the three-rater and four-rater solutions (Supplementary File 12; mean correlation between component scores, *r* = 0.89). All the analyses were performed in R version 4.4.1 (Supplementary File 13–17)^84^.

## Supplementary Information

Below is the link to the electronic supplementary file.

Supplementary File 8

Supplementary File 7

Supplementary File 6

Supplementary File 5

Supplementary File 12

Supplementary File 1

Supplementary File 3

Supplementary File 2

Supplementary File 10

Supplementary File 9

Supplementary File 4

Supplementary File 11

Supplementary File 13

Supplementary File 14

Supplementary File 15

Supplementary File 16

Supplementary File 17

## Data Availability

The datasets are available in the supplementary file.
